# Change in Computed Tomography-Derived Fractional Flow Reserve Across the Lesion Improve the Diagnostic Performance of Functional Coronary Stenosis

**DOI:** 10.3389/fcvm.2021.788703

**Published:** 2022-01-13

**Authors:** Hankun Yan, Yang Gao, Na Zhao, Wenlei Geng, Zhihui Hou, Yunqiang An, Jie Zhang, Bin Lu

**Affiliations:** Department of Radiology, Fu Wai Hospital, National Center for Cardiovascular Diseases, Peking Union Medical College, Chinese Academy of Medical Sciences, Beijing, China

**Keywords:** coronary artery disease, fractional flow reserve, coronary computed tomography angiography, computed tomography-derived fractional flow reserve, machine-learning

## Abstract

**Aims:** This study sought to evaluate the diagnostic performance of change in computed tomography-derived fractional flow reserve (CT-FFR) across the lesion (ΔCT-FFR) for identifying ischemia lesions with FFR as the reference standard.

**Methods:** Patients who underwent coronary CT angiography (CCTA) and FFR measurement within 1 week from December 2018 to December 2019 were retrospectively enrolled. CT-FFR within 2 cm distal to the lesion, ΔCT-FFR and plaque characteristics were analyzed. The diagnostic accuracy of CCTA (coronary stenosis ≥ 50%), CT-FFR ≤ 0.80, and ΔCT-FFR ≥ 0.15 (based on the largest Youden index) were assessed with FFR as the reference standard. The relationship between plaque characteristics and ΔCT-FFR was analyzed.

**Results:** The specificity of ΔCT-FFR and CT-FFR were 70.8 and 67.4%, respectively, which were both higher than CCTA (39.3%) (both *P* < 0.001), while there were no statistical significance in sensitivity among the three (84.5, 77.4, 88.1%, respectively; *P* = 0.08). The area under the curves (AUCs) of ΔCT-FFR and CT-FFR were 0.803 and 0.743, respectively, which were both higher than that of CCTA (0.637) (both *P* < 0.05), and the AUC of ΔCT-FFR was higher than that of CT-FFR (*P* < 0.001). Multivariable analysis showed that low-attenuation plaque (LAP) volume (odds ratio [OR], 1.006) and plaque length (OR, 1.021) were independently correlated with ΔCT-FFR (both *P* < 0.05).

**Conclusions:** CT-FFR and ΔCT-FFR and here especially the ΔCT-FFR could improve the diagnostic performance of ischemia compared with CCTA alone. LAP volume and plaque length were the independent risk factors of ΔCT-FFR.

## Introduction

Considering the limitations of coronary computed tomography angiography (CCTA) in the diagnosis of ischemic lesions and the importance of invasive fractional flow reserve (FFR) physiological evaluation in guiding clinical treatment (1–3), non-invasive computed tomography-derived FFR (CT-FFR) has attracted more and more attention since its emergence. CT-FFR is a non-invasive image post-processing technology. Several studies have shown that CT-FFR has good diagnostic performance, and it also has been proved to be highly correlated with invasive FFR (4–6). Recently ACC/AHA published guidelines for chest pain where CCTA was given 1A status for the evaluation of intermediate-risk patients with acute chest pain and no known CAD, and CT-FFR to a class 2a recommendation with a B-NR level of evidence (7). At present, the calculation methods of CT-FFR mainly include the computational fluid dynamics method and machine-learning (ML) method, and both methods have good diagnostic performance, but the ML method requires shorter calculation time and computational power (8, 9). Several previous studies have analyzed different measurement positions of CT-FFR, suggesting that CT-FFR should be measured at the distal to the lesion rather than to the vessel (10–12). Compared with CT-FFR distal to the lesion, Takagi et al. (13) found the difference between CT-FFR proximal and distal to the lesion (ΔCT-FFR) had higher diagnostic performance (area under the curve [AUC]: 0.86 vs. 0.71, *P* < 0.01). However, the sample size of this study is so small that its conclusion needs to be verified by larger sample size. Previous studies have shown plaque characteristics predict lesion-specific ischaemia (14, 15). However, there is no study to analyze the relationship between plaque characteristics and ΔCT-FFR of the lesion vessel. The purpose of this study was to explore the diagnostic performance of ΔCT-FFR and CT-FFR distal to the lesion and analyze the relationship between plaque characteristics and ΔCT-FFR.

## Materials and Methods

### Study Population

A retrospective collection of consecutive patients with suspected or known CAD who underwent CCTA, ICA and invasive FFR measurement within 1 week from December 2018 to December 2019 were enrolled in this study. The inclusion criteria were as follows: (1) age ≥ 18 years; (2) there was at least one lesion with stenosis degree between 30 and 90% on CCTA. Exclusion criteria included: (1) previous history of myocardial infarction and/or coronary revascularization; (2) the quality of CT image was too poor to extract the coronary artery tree for CT-FFR. Approval for the study was obtained from the Institutional Review Board of our hospital (IRB approval number: NO.2018-1076), and the patient consent was waived because the study has retrospective nature.

### CCTA Acquisition

All patients in this study underwent CCTA with dual source CT scanner (Definition Flash, Siemens Healthcare, Forchheim, Germany). Image acquisition was performed according to the cardiovascular computed tomography protocol (16). All patients were scanned by prospective electrocardiogram (ECG) gating technology, and images were acquired at 35–75% of R-R interval. The heart rate of all patients was controlled below 75 beats/min. Patients would be given beta-blocker sublingually before the examination if the heart rate is > 75 beats/min, and scan again when the heart rate drops below 75 beats/min. The scanning parameters were shown as follows: tube voltage, 100 or 120 kV, tube current, automatic tube current modulation; rotation time, 0.28 s per rotation; Slice thickness, 0.75 mm; increment 0.70 mm. The raw CT data were reconstructed by use of iterative reconstruction with filtering, and the optimal cardiac phase with the minimum motion artifact was determined by radiologic technicians. Briefly, 60–70 ml contrast medium (Iohexol, Shuangbei 350; Beilu Pharmaceutical Co., Ltd., Beijing, China) was injected into antecubital vein at 4.5–5.0 ml/s via a dual-cylinder high-pressure syringe (Stellant; Medrad, Indianola, Pennsylvania), followed by a 30–40 ml saline flush at the same rate.

### Coronary Stenosis and Plaque Analysis

Coronary artery calcification was scored according to Agatston et al. (17). The CT images were analyzed by two senior doctors who did not know the patients' condition, and the degree of stenosis was graded according to the percentage diameter stenosis (%DS) of the target lesion: mild stenosis (30–49%), moderate stenosis (50–69%), severe stenosis (70–90%). For those with different opinions, the final result will be obtained after discussion. Coronary artery stenosis ≥ 50% was considered as obstructive stenosis.

Coronary plaque was analyzed by using semi-automatic post-processing software (QAngio CT Research Edition v3.0; Medis medical imaging systems, Leiden, The Netherlands). Plaques with area > 1 mm^2^ in coronary lumen with diameter ≥ 2 mm were analyzed. Plaque components include three parts: low-attenuation plaque (LAP) (attenuation < 30 Hounsfield units [HU]), intermediate-attenuation plaque (IAP) (attenuation between 30 and 130 HU) and calcification component (attenuation > 130 HU). The quantification of each plaque component is automatically generated according to the specific attenuation threshold in the manually specified area. Two doctors with more than 5 years of clinical experience used the post-processing workstation to analyze the plaque at the vascular level independently without knowing the specific condition of the patients, and the average value was used for analysis. Relevant parameters were recorded, including the total plaque volume, the volume of each plaque component of each lesion vessel, as well as the plaque length of the target lesion plaque at the most severe vascular stenosis. At the same time, four characteristics of high-risk plaques are analyzed, which are effective predictors of poor prognosis (18, 19). The remodeling index is the ratio of the maximum vessel diameter at the lesion site to the vessel diameter at the proximal reference point, and the remodeling index > 1.1 indicates positive remodeling (PR) (14). LAP is defined as a plaque containing components with a density lower than 30 HU (14). Spotty calcification (SC) is characterized by visible tiny calcified plaque (< 90° vessel circumference, diameter < 3 mm) (14). The napkin ring sign (NRS) is defined as an area with a low attenuation in the center and a higher attenuation around the edges (19). It can be defined as a high-risk plaque when there are at least two or more of the above-mentioned plaque characteristics.

### CT-FFR Acquisition and Analysis

This research used ML-based software (cFFR 3.0, Siemens Healthineers, Forchheim, Germany) to conduct CT-FFR. The research software is not yet commercially available. Itu et al. (20) have reported detailed information on the basic principles of the CT-FFR calculation of this method previously. The value of CT-FFR was measured within 2 cm proximal and distal to the lesion plaque (CT-FFR_proximal_, CT-FFR_distal_), respectively, and CT-FFR_distal_ ≤ 0.80 was considered to be an ischemic lesion. All measurement positions of CT-FFR were co-located with invasive FFR. Then the difference between CT-FFR_proximal_ and CT-FFR_distal_ was calculated to obtain the change in CT-FFR across the lesion (ΔCT-FFR), as shown below: ΔCT-FFR = CT-FFR_proximal_ - CT-FFR_distal_. In order to evaluate the reproducibility between observers, two radiologists (with 5 and 8 years of work experience, respectively), completed the CT-FFR measurements of 30 consecutive vessels without knowing the patients' condition, independently.

### ICA and FFR Measurement

Invasive FFR measurement was completed during the ICA inspection, and all operations were performed by senior cardiovascular physicians with rich work experience. The standard posture of each patient was taken for inspection, and at least 2 different angles for each main vessel were selected for observation. FFR measurement was performed according to the method reported in the past literature (21): FFR was the ratio of the pressure of the distal coronary artery (measured by the pressure guide wire) divided by the aortic pressure (measured by the guide catheter) during the maximum congestion period. FFR was measured by using 0.014 inch pressure guide wire (St Jude Medical Systems, Minneapolis, USA). Pressure guide wire was placed at the end of the guide tube to calibrate itself. To measure the FFR value, the pressure wire was located distally to the lesion about 2 cm and maximal hyperaemia state was induced by continuous intravenous infusion of adenosine (160 μg/kg/min). Subsequently, the pressure guide wire was pulled back slowly from the distal part of the lesion vessel to the proximal part during induced steady-state maximal hyperemia to confirm the consistency of the two pressure values. Invasive FFR ≤ 0.8 was considered that vessel stenosis was hemodynamically significant (1, 2).

### Statistical Analysis

Continuous data were presented as mean ± standard deviation (SD) in case of normal distribution, median (interquartile range) in case of non-normal distribution, and categorical data were expressed as numbers and percentages. To evaluate interobserver reproducibility, Cohen's Kappa statistic was used to analyze the consistency of CCTA diagnosis results of the two doctors, and intraclass correlation coefficients were used to evaluate the inter-observer variability of CT-FFR and ΔCT-FFR. The receiver operator characteristic curve (ROC) was created to predict the area under the curve (AUC), *P* values, diagnostic accuracy, sensitivity, specificity, positive predictive value (PPV), and negative predictive value (NPV), by using invasive FFR as the gold standard. The AUCs of different methods were compared as previously described by Delong et al. (22). The diagnostic accuracy, sensitivity and specificity of different methods were compared by Cochran's Q test, then the post Dunn test and Bonferroni correction were used for inter group comparison (23), and chi-square was used to compare positive predictive value (PPV) and negative predictive value (NPV). The best cut-off value of ΔCT-FFR was selected according to the largest Youden index (defined as %sensitivity +%specificity - 1). All vessels were divided into two groups by taking the cut-off value of ΔCT-FFR, and Student's *t*-test and chi-square test were performed to compare the data. The relationship between plaque characteristics and ΔCT-FFR was analyzed by binary logistic regression. All statistical analyses were performed with SPSS 25.0 and Medcalc 19.0.4. All statistical tests were two-tailed. *P* < 0.05 indicated that the difference was statistically significant.

## Results

### Patient Characteristics

**Figure 1:**
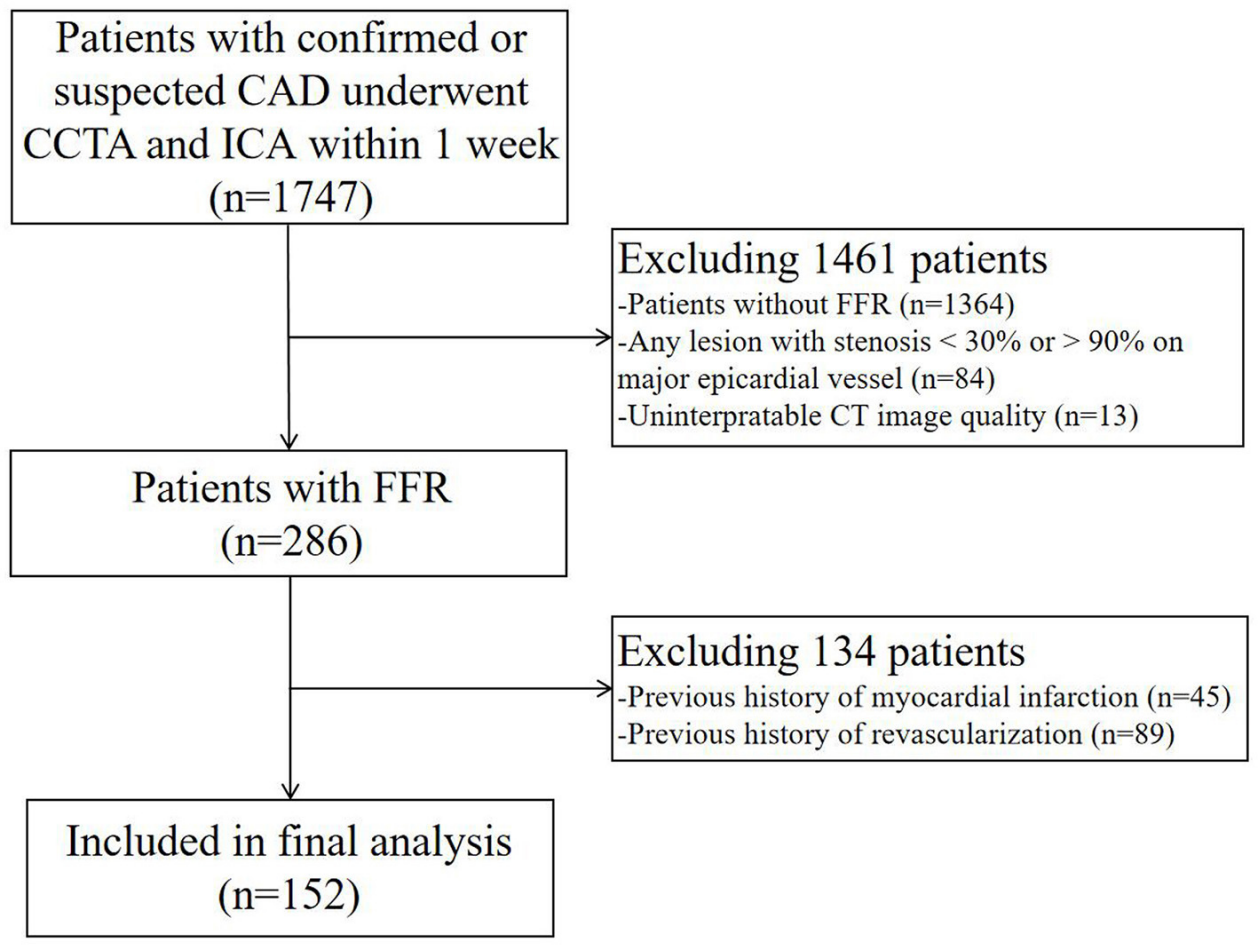
Flowchart of patient selection. CCTA, coronary computed tomography angiography; ICA, invasive coronary angiography; FFR, fractional flow reserve; CT, computed tomography.

There were 152 patients included in this study. The patient selection process is shown in Figure 1. The baseline data of patients are shown in Tables 1, 2. The mean age of the patients was 56.6 ± 9.1 years. There were 115 (75.7%) males and 37 (24.3%) females. 21 (13.8%) patients with calcification score ≥ 400 and 21 (13.8%) patients had multiple lesion vessels. Among the 173 vessels, left anterior descending coronary artery (LAD), left circumflex coronary artery (LCX), and right coronary artery (RCA) accounted for 121 (69.9%), 27 (15.6%), and 25 (14.5%) of the total lesion vessels, respectively; there were 128 (74.0%) vessels with obstructive stenosis (moderate and severe stenosis) on CCTA and 84 (48.6%) vessels with invasive FFR ≤ 0.80.

**Table 1:** Patient characteristics.

**Characteristic**	**Value**
**Patient characteristic**	
Number of patients, n	152
Number of Lesion Vessels, n	173
Age, year	56.6 ± 9.1
**Sex**	
Male, n (%)	115 (75.7)
Female, n (%)	37 (24.3)
BMI, kg/m^2^	26.14 ± 3.09
**Cardiovascular risk factors**	
Hypertension, n (%)	91 (59.9)
Diabete, n (%)	52 (34.2)
Dyslipidemia, n (%)	128 (84.2)
Current/past smoker, n (%)	82 (53.9)
Family history of CAD, n (%)	20 (13.2)
**Medications at baseline**	
Aspirin, n (%)	141 (92.8)
Beta-blocker, n (%)	14 (9.2)
Calcium-channel blocker, n (%)	71 (46.7)
Statins, n (%)	130 (85.5)

*Continuous variables were expressed as mean ± standard deviation values, and categoric variables were expressed as numbers (percentages) of patients or lesions. BMI, body mass index; CAD, coronary artery disease*.

**Table 2:** Lesion and CT characteristics.

**Characteristic**	**Value**
**Lesion characteristics**	
**Vessel assessed**	
LAD, n (%)	121 (69.9)
LCX, n (%)	27 (15.6)
RCA, n (%)	25 (14.5)
Agatston score	69.0 (11.3–234.8)
<400, n (%)	131/152 (86.2)
≥ 400, n (%)	21/152 (13.8)
**Vessel with CCTA maximum stenosis**	
Mild stenosis, n (%)	45 (26.0)
Moderate stenosis, n (%)	60 (34.7)
Severe stenosis, n (%)	68 (39.3)
**Invasive FFR**	0.81 (0.70–0.87)
Vessels with FFR ≤ 0.80, n (%)	84 (48.6)
RCA with FFR ≤ 0.80, n (%)	7 (4.1)
LAD with FFR ≤ 0.80, n (%)	69 (39.9)
LCx with FFR ≤ 0.80, n (%)	8 (4.6)
Patients with multivessel disease, n (%)	21 (13.8)
**CT characteristics**	
Heart rate, beats/min	66 (59–75)
DLP for CCTA, mGy·cm	489.0 (376.0–665.5)
Effective radiation dose for CCTA, mSv	6.9 (5.3–9.3)
**High-risk plaque, n (%)**	30 (17.3)
PR, n (%)	21 (12.1)
LAP, n (%)	24 (13.9)
SC, n (%)	54 (31.2)
NRS, n (%)	31 (17.9)

*Continuous variables were expressed as median (interquartile range) values, and categoric variables were expressed as numbers (percentages) of patients or lesions. Not all percentages total 100% because of rounding*.

*LAD, left anterior descending coronary artery; LCX, left circumflex coronary artery; RCA, right coronary artery; CCTA, coronary computed tomography angiography; FFR, fractional flow reserve; DLP, dose-length product; PR, positive remodeling; SC, spotty calcification; LAP, low-attenuation plaque; NRS, napkin ring sign*.

### Diagnostic Performance of Different Methods

The kappa value of CCTA was 0.813 (95% CI, 0.759–0.871), *P* < 0.001. The intraclass correlation coefficients were shown as follows: ΔCT-FFR, 0.98 (95% CI, 0.94–0.99); CT-FFR, 0.97 (95% CI, 0.93–0.99).

**Figure 2:**
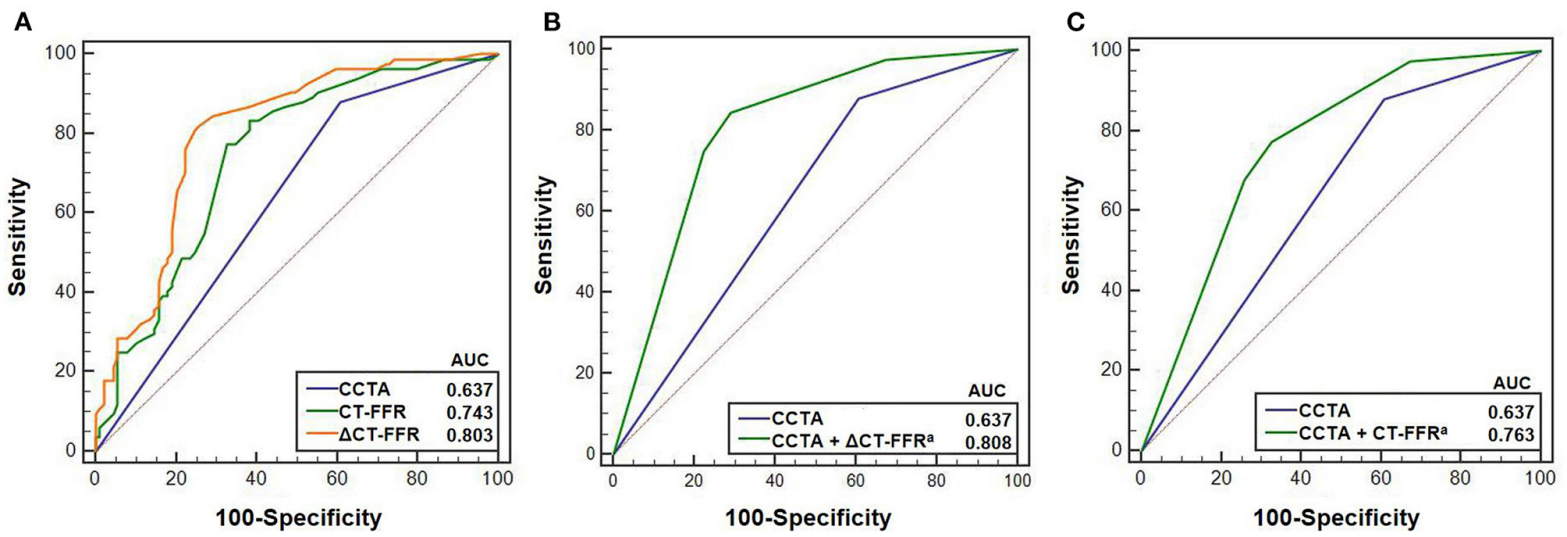
Receiver operating characteristic (ROC) curves of CCTA, ΔCT-FFR, CT-FFR in predicting ischemia (*N* = 173 vessels). **(A)** shows ROC curves for predicting ischemia using CCTA, ΔCT-FFR and CT-FFR. **(B,C)** show the ROC curves of models using CCTA with and without ΔCT-FFR and CT-FFR, respectively. Cut-off value of 0.15 corresponding to the maximum Youden index was used for the comparison between CCTA with and without ΔCT-FFR. ^a^Indicates there were statistically significant difference between AUC for CCTA and CCTA with ΔCT-FFR and CT-FFR, respectively **(B,C)** using DeLong test (24). CCTA, coronary computed tomography angiography; CT-FFR, computed tomography-derived fractional flow reserve; AUC, area under curve.

The AUCs of ΔCT-FFR, CT-FFR, and CCTA were 0.803 (95% CI, 0.736–0.859), 0.743 (95% CI, 0.672–0.807), 0.637 (95% CI, 0.561–0.709), respectively (Figure 2). The best cut-off value of ΔCT-FFR was 0.15 according to the largest Youden index.

**Figure 3:**
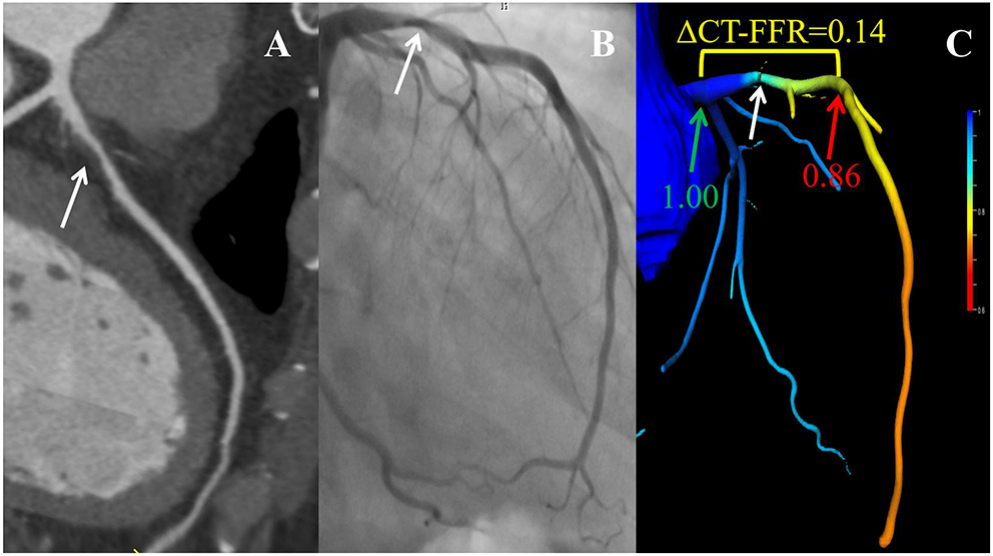
Example of a 56-year-old man with chest pain. CCTA **(A)** and ICA **(B)** as well as CT-FFR **(C)** showing an LAD with moderate stenosis (white arrow). ΔCT-FFR was the difference of values obtained by subtracting CT-FFR_distal_ (red arrow) from CT-FFR_proximal_ (green arrow), where CT-FFR_proximal_ and CT-FFR_distal_ were defined as the values proximal or distal within 2 cm to the lesion plaque, respectively. CCTA, coronary computed tomography angiography; ICA, invasive coronary angiography; CT-FFR, computed tomography-derived fractional flow reserve; LAD, left anterior descending coronary artery.

The AUC of ΔCT-FFR was higher than other methods: difference in AUC for CT-FFR was 0.060 (95% CI, 0.037–0.082, *P* < 0.001), and CCTA was 0.166 (95% CI, 0.083–0.248, *P* < 0.001). The difference in AUC of CT-FFR was 0.106 (95% CI, 0.020–0.193, *P* = 0.016) higher than CCTA. The diagnostic characteristics of the three methods are shown in Table 3. The accuracy of ΔCT-FFR and CT-FFR were both higher than CCTA (*P* < 0.001, *P* = 0.049, respectively), and specificity as well (both *P* < 0.001), while there were no statistically significant difference between the accuracy and specificity of ΔCT-FFR and CT-FFR (*P* = 0.534, *P* = 1.000, respectively). The PPV of ΔCT-FFR was higher than that of CCTA (*P* = 0.017), and there were no statistically significant difference between ΔCT-FFR and CT-FFR (*P* = 0.537); there were no statistically significant differences in NPV and sensitivity among them (*P* = 0.555, *P* = 0.08). Figure 3 shows a representative case of patients with moderate stenosis (50–69%) without hemodynamically significant stenosis (invasive FFR = 0.88).

**Table 3:** Per-vessel diagnostic accuracy of ΔCT-FFR, CT-FFR, and CCTA.

	**True** **positive**^**a**^	**True** **negative**^**a**^	**False** **positive**^**a**^	**False** **negative**^**a**^	**%** **Accuracy**	**%** **Sensitivity**	**%** **Specificity**	**%** **PPV**	**%** **NPV**	**AUC**
ΔCT-FFR	71	63	26	13	77.5 (70.6–83.1)	84.5 (75.0–91.5)	70.8 (60.2–80.0)	73.2 (66.1–0.79.3)	82.9 (74.3–89.1)	0.803 (0.736–0.859)
CT–FFR	65	60	29	19	72.3 (65.1–78.4)	77.4 (67.0–85.8)	67.4 (56.7–77.0)	69.2 (61.9–75.5)	76.0 (67.5–82.8)	0.743 (0.672–0.807)
CCTA	74	35	54	10	63.0 (55.6–70.0)	88.1 (79.2–94.1)	39.3 (29.1–50.3)	57.8 (53.3–62.2)	77.8 (64.9–86.9)	0.637 (0.561–0.709)

*Except otherwise indicated, data are percentage with 95% confidence intervals*.

a *Data are raw data*.

*CT-FFR, computed tomography-derived fractional flow reserve; CCTA, coronary computed tomography angiography; PPV, positive predictive value; NPV, negative predictive value; AUC, area under curve; CI, confidence interval*.

### Additive Values of CT-FFR

Compared with the model only using CCTA, both diagnostic models using CCTA with ΔCT-FFR or CT-FFR could obtain a larger AUC (CCTA, 0.637, 95% CI, 0.561–0.709; CCTA + ΔCT-FFR, 0.808, 95% CI, 0.741–0.864, *P* < 0.001; CCTA + CT-FFR, 0.763, 95% CI, 0.693–0.825, *P* = 0.001) (Figure 2).

### Relationship Between Coronary Stenosis, CT-FFR and ΔCT-FFR

**Figure 4:**
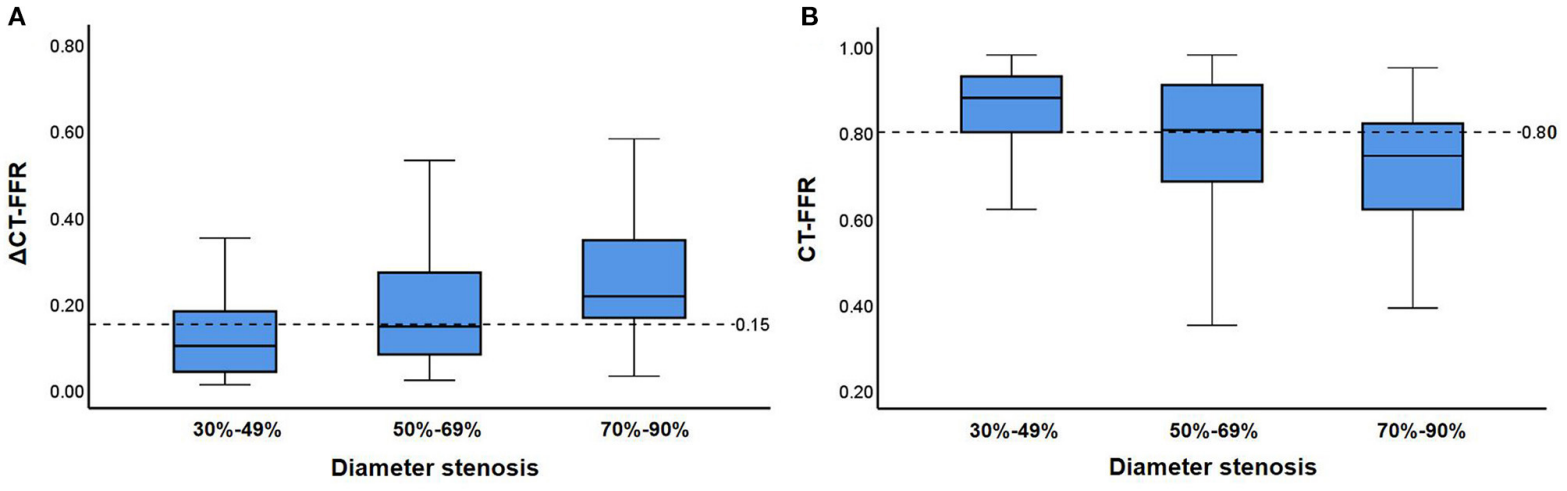
Relationship between CT-FFR and stenosis on CCTA. **(A,B)** show distributions of ΔCT-FFR **(A)**, CT-FFR **(B)** in each group with 30–49, 50–69, and 70–90% diameter stenosis on CCTA. Medians, quartiles, and ranges of ΔCT-FFR as well as CT-FFR are shown in the box plot. Cut-off values of ΔCT-FFR as well as CT-FFR are displayed as dashed lines. CT-FFR, computed tomography-derived fractional flow reserve.

The relationship between anatomical stenosis determined by CCTA, CT-FFR and ΔCT-FFR was shown in Figure 4. Among the 128 vessels with obstructive stenosis (≥ 50%), ΔCT-FFR ≥ 0.15 accounted for 64.8% (83/128), while CT-FFR ≤ 0.80 accounted for 62.5% (80/128). Among 68 severe stenosis (70–89%) lesions in CCTA, 47 (69.1%) were demonstrated with hemodynamic significance (invasive FFR ≤ 0.8); while moderate stenosis (50–69%) and mild stenosis (30–49%) accounted for 45.0% (27/60) and 22.0% (10/45), respectively. For severe stenosis (70–89%) lesions, both ΔCT-FFR and CT-FFRT could reclassify 14.7% (10/68) lesions as non-ischemic (invasive FFR > 0.80); for moderate stenosis (50–69%) lesions, ΔCT-FFR or CT-FFR could correctly reclassify 40.0% (24/60) and 35.0% (21/60) lesions as non-ischemic (invasive FFR > 0.80), respectively; for mild stenosis (30–49%) lesions, ΔCT-FFR and CT-FFR could both correctly reclassify 17.8% (8/45) lesions as ischemic (invasive FFR ≤ 0.80). In this study, there were 34 CT-FFR values of lesion vessels in the gray zone (0.75-0.80), of which 10 (29.4%) vessels had invasive FFR > 0.80. And ΔCT-FFR can correctly classify 30% (3/10) lesions as non-ischemic (invasive FFR > 0.80).

### Relationship Between Plaque Characteristics and ΔCT-FFR

As shown in Table 4, 83 (85.6%) vessels with ΔCT-FFR ≥ 0.15 and 45 (59.2%) vessels with ΔCT-FFR < 0.15 had obstructive stenosis (*P* < 0.001). The LAP volume and plaque length of the patients of the ΔCT-FFR ≥ 0.15 group were higher than those of the ΔCT-FFR < 0.15 group (*P* = 0.005, *P* = 0.003, respectively). The results of logistic regression are shown in Table 5. In Univariable analysis, LAP volume (OR, 1.008, 95% CI, 1.002–1.014, *P* = 0.005) and plaque length (OR, 1.028, 95% CI, 1.007–1.050, *P* = 0.009) were related to ΔCT-FFR. In multivariable analysis, both LAP volume (OR, 1.006, 95% CI, 1.001–1.012, *P* = 0.028) and plaque length (OR, 1.021, 95% CI, 1.000–1.043, P < 0.048) remains the correlation with ΔCT-FFR.

**Table 4:** Coronary stenosis severity and plaque characteristics according to ΔCT-FFR (ΔCT-FFR ≥ 0.15).

	**ΔCT-FFR**		***P***
	** <0.15** **(*n* = 76)**	**≥ 0.15** **(*n* = 97)**	
Stenosis ≥ 50%^a^, n (%)	45 (59.2)	83 (85.6)	<0.001
High-risk plaque^a^, n (%)	9 (11.8)	21 (21.6)	0.091
Total plaque volume, mm^3^	335.1 ± 16.9	359.9 ± 22.7	0.406
LAP volume, mm^3^	67.4 ± 53.1	99.0 ± 85.5	0.005
IAP volume, mm^3^	234.1 ± 108.4	218.1 ± 121.0	0.366
Calcification volume, mm^3^	26.2 ± 33.5	38.0 ± 81.1	0.195
Plaque length, mm	23.8 ± 12.4	31.8 ± 22.4	0.003
Agatston score of lesion vessel	19.4 ± 29.6	34.4 ± 80.5	0.093

*Except otherwise indicated, data are mean ± SD*.

a *Data are number (percentage)*.

*CT-FFR, computed tomography-derived fractional flow reserve; LAP, low-attenuation plaque; IAP, intermediate-attenuation plaque*.

**Table 5:** Univariable and multivariable analysis of plaque characteristics for prediction of ΔCT-FFR (ΔCT-FFR ≥ 0.15).

	**Univariable analysis**		**Multivariable analysis**	
	**OR (95% CI)**	***P***	**OR (95% CI)**	***P***
High-risk plaque	0.486 (0.208–1.134)	0.095	-	-
Total plaque volume	1.001 (0.999–1.002)	0.408	-	-
LAP volume	1.008 (1.002–1.014)	0.005	1.006 (1.001–1.012)	0.028
IAP volume	0.999 (0.996–1.001)	0.368	-	-
Calcification volume	1.003 (0.998–1.009)	0.253	-	-
Plaque length	1.028 (1.007–1.050)	0.009	1.021 (1.000–1.043)	0.048
Agatston score of lesion vessel	1.001 (1.000–1.003)	0.142	-	-

*OR, odds ratio; CI, confidence interval; LAP, low-attenuation plaque; IAP, intermediate-attenuation plaque*.

## Discussion

The main findings of this study were: (1) compared with CCTA, ΔCT-FFR and CT-FFR have higher diagnostic AUC and accuracy, among which ΔCT-FFR has the highest diagnostic performance; (2) both ΔCT-FFR and CT-FFR can reclassify hemodynamic significant lesions effectively, and ΔCT-FFR can improve the recognition of gray zone lesions of CT-FFR; (3) LAP volume and plaque length were the independent risk factors of functional ΔCT-FFR after adjusting for confounding factors.

A major challenge in the clinical application of CT-FFR is the measurement method of CT-FFR. Unlike invasive FFR, CT-FFR measured at the distal to the vessel tends to overestimate ischemic lesions (24, 25). In order to standardize the clinical application of CT-FFR, some experts recommended that the CT-FFR value should be measured within 2 cm distal to the lesion rather than at the nadir or distal (26, 27). However, previous studies (25) have shown that regardless of whether there is stenosis, the value of CT-FFR will gradually decrease along the long axis of the lumen. There would be differences between CT-FFR and invasive FFR measured at different locations. ΔCT-FFR reflects the change value of CT-FFR proximal and distal to the specific lesion, and shows the change of hemodynamics of the lesion directly. Studies have shown that ΔCT-FFR could improve the ability to recognize lesions that cause ACS (28), and have a better diagnostic performance than CT-FFR distal to the lesion (13). In this study, both ΔCT-FFR and CT-FFR have higher AUC and accuracy than CCTA, among which ΔCT-FFR had the highest AUC, and when the cut-off value is 0.15, ΔCT-FFR has the largest Youden index. The ΔCT-FFR sensitivity (84%) of per-vessel was consistent with the NXT test (6), but the specificity was lower than that of the NXT test (86%), significantly. The reasons might be related to the difference in the sample sizes of the two studies and the CT-FFR analysis software. Nevertheless, in this study, the specificity of ΔCT-FFR (71%) was significantly higher than that of CCTA (39%) while maintaining high NPV (83%, 78%, respectively). This study suggested that ΔCT-FFR might improve the treatment strategy of patients, which could reduce unnecessary downstream examination and costs (29).

Similar to previous study (29), more than half of the vessels with obstructive stenosis had ΔCT-FFR ≥ 0.15 or CT-FFR ≤ 0.8. The results of this study showed that both ΔCT-FFR and CT-FFR could reclassify vessels with moderate stenosis effectively. ΔCT-FFR could classify nearly half of vessels with moderate stenosis as non-ischemic vessels correctly; and both ΔCT-FFR and CT-FFR had partial reclassification ability in mild and severe stenosis. Combining ΔCT-FFR or CT-FFR could help to improve the correct diagnosis of ischemic lesions on the basis of the anatomical information provided by CCTA. Besides, lesions in gray zone always trouble the clinical diagnosis and treatment of patients, and our study showed ΔCT-FFR could improve the recognition of gray zone lesions of CT-FFR, which might mean that ΔCT-FFR is more suitable for clinical application than CT-FFR. However, a large sample study is still needed for verification.

Several previous studies have confirmed that there was an association between coronary atherosclerotic plaque characteristics and hemodynamically significant ischemia (14, 15, 30). As a non-invasive alternative to invasive FFR, CT-FFR should maintain the correlation with plaque characteristics. Compared with patients with ΔCT-FFR < 0.15, this study found for the first time that, patients with ΔCT-FFR ≥ 0.15 had higher LAP volume and plaque length, and both them were the independent risk factor of ΔCT-FFR, which were in accordance with the relationship between invasive FFR and plaque characteristics of previous results (15). LAP is the surrogate of necrotic core (31). Studies have shown that plaques with necrotic core would cause local inflammation and oxidative stress, which would lead to local vascular endothelial dysfunction and local “functional stenosis” (32), and it was the main cause of myocardial infarction and sudden cardiovascular death (33, 34). And at the mean time, we should pay attention to those long-length lesions with non-obstructive stenosis, which might could cause hemodynamically significant ischemia. The high-risk plaques in this study showed no significant difference between the two groups with ΔCT-FFR = 0.15 as the cut-off value, which might be because the small sample size of high-risk plaques in this study (*N* = 30).

There are some limitations in this study. This study was a retrospective, single-center study. This study included only those patients with stable chest pain who had undergone ICA and invasive FFR assessments in the cath lab and had not undergone coronary revascularization surgery. Patients with the acute coronary syndrome were excluded from the study, which might lead to potential selection bias. Besides, this study lacks clinical outcome data. Therefore, further clinical outcome studies are still needed to analyze the effectiveness of these methods.

## Conclusions

CT-FFR and especially ΔCT-FFR were additional tools to identify patients with relevant stenosis and both tools and here especially the ΔCT-FFR could improve the diagnostic performance of ischemia compared with CCTA alone. Thus, the need for further invasive treatment could be better applied to patients. LAP volume and plaque length were the independent risk factors of ΔCT-FFR.

## Data Availability Statement

The original contributions presented in the study are included in the article/supplementary material, further inquiries can be directed to the corresponding author/s.

## Ethics Statement

The studies involving human participants were reviewed and approved by the Ethics Committee of Fuwai Hospital, National Center for Cardiovascular Diseases. Written informed consent for participation was not required for this study in accordance with the national legislation and the institutional requirements.

## Author Contributions

HY, YG, ZH, and BL conception and design and administrative support. HY, NZ, and WG collection and upload of data. HY, JZ, and YA data analysis and interpretation. HY wrote the first draft of the paper. YG and BL revised the article. All authors contributed to the article and approved the submitted version.

## Funding

This study was supported by the Clinical and Translational Medicine Research Foundation of Chinese Academy of Medical Sciences (2019XK320065) and the Ministry of Science and Technology of China, National Key Research and Development Project (2016YFC1300402).

## Conflict of Interest

The authors declare that the research was conducted in the absence of any commercial or financial relationships that could be construed as a potential conflict of interest.

## Publisher's Note

All claims expressed in this article are solely those of the authors and do not necessarily represent those of their affiliated organizations, or those of the publisher, the editors and the reviewers. Any product that may be evaluated in this article, or claim that may be made by its manufacturer, is not guaranteed or endorsed by the publisher.
